# Humoral immunogenicity of ChAd63_MVA ME-TRAP vaccination in African infants and children

**DOI:** 10.1186/1475-2875-13-S1-P16

**Published:** 2014-09-22

**Authors:** Georgina Bowyer, Muhammed O Afolabi, Carly M Bliss, Alfred B Tiono, Abdoulie Drammeh, Issa Nébié, Ya Jankey Jagne, Jean Baptiste Yaro, Egeruan B Imoukhuede, Katie L Flanagan, Beate Kampman, Nicola Viebig, Sodiomon B Sirima, Kalifa Bojang, Adrian VS Hill, Katie J Ewer

**Affiliations:** 1 Jenner Institute Laboratories, University of Oxford, Oxford, UK; 2 Medical Research Council Unit, Fajara, Gambia; 3 Centre National de Recherche et de Formation sur le Paludisme, Ouagadougou, Burkina Faso; 4 Centre for Clinical Vaccinology and Tropical Medicine, Oxford, UK; 5 European Vaccine Initiative, Heidelberg, Germany

## Background

The only malaria vaccine to have been tested in Phase III clinical vaccine trials [1] induces protection which has been associated with high titres of antibodies against sporozoites. Efficacy against clinical malaria in infants of 5-17 months and 6-12 weeks was 55.8% and 31.3% respectively. A successful malaria vaccine will likely need to induce both cellular and humoral immunity in order to achieve a deployable level of efficacy in the target age groups. Prime-boost immunisation with viral vectors ChAd63 and MVA, both encoding ME-TRAP, has been shown to induce high T cell responses and modest antibody responses to the pre-erythrocytic malaria antigen TRAP. A Phase II study with this vaccine regime in malaria-naïve adults showed significant efficacy, which correlated with frequency of monofunctional CD8+ T cells secreting IFNγ. TRAP antibody titres were modest and did not correlate with protection [2]. In contrast to this, high titres of vaccine-induced anti-TRAP antibodies are measured in infants in malaria-endemic settings.

## Materials and methods

Antibody titres were measured in 138 malaria-exposed children vaccinated with ChAd63 MVA ME-TRAP in three Phase I studies in The Gambia and Burkina Faso. Age groups at first immunisation were 2-6 years, 5-12 months and 10 weeks in The Gambia and 5-17 months in Burkina Faso. Avidity and isotype profiles were also analyzed.

## Results

Antibody responses to TRAP were significantly higher in 10 week old and 5-12 month old infants in The Gambia and 5-17 month old infants in Burkina Faso compared to 2-6 year old children and adults in The Gambia and malaria-naïve UK adults. IgG isotype responses were predominantly IgG1 and IgG3 and we also detected IgA and IgM. TRAP-specific IgG avidity was significantly higher in Burkinabe infants aged 5-17 months and Gambian infants aged 5-12 months compared to Gambian adults and 2-6 year old children. TRAP-specific IgG1 avidity significantly correlated with age at vaccination in 5-17 month old Burkinabe infants and was significantly higher than in 10 week old Gambian infants. Functional activity of anti-TRAP antibodies will be analysed *in vitro* using a sporozoite invasion inhibition assay.

## Conclusions

We demonstrate excellent humoral immunogenicity in key target populations vaccinated with a pre-erythrocytic malaria vaccination regime, exceeding that seen in UK malaria-naïve adults.
